# Study of Monoclonal Antibody Aggregation at the Air–Liquid Interface under Flow by ATR-FTIR Spectroscopic Imaging

**DOI:** 10.1021/acs.langmuir.3c03730

**Published:** 2024-03-06

**Authors:** Céline van Haaren, Bernadette Byrne, Sergei G. Kazarian

**Affiliations:** † Department of Chemical Engineering, Imperial College London, South Kensington Campus, London SW7 2AZ, U.K.; ‡ Department of Life Sciences, Imperial College London, South Kensington Campus, London SW7 2AZ, U.K.

## Abstract

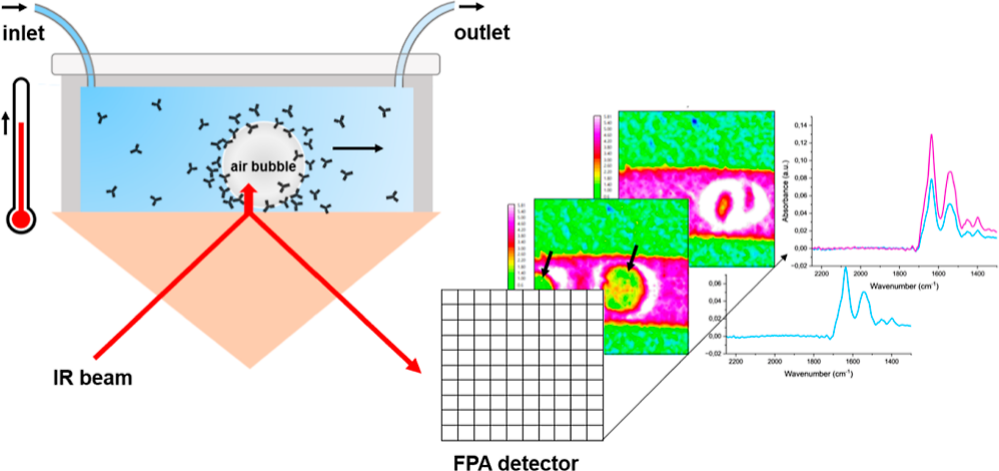

Throughout bioprocessing, transportation, and storage, therapeutic monoclonal antibodies (mAbs) experience stress conditions that may cause protein unfolding and/or chemical modifications. Such structural changes may lead to the formation of aggregates, which reduce mAb potency and may cause harmful immunogenic responses in patients. Therefore, aggregates need to be detected and removed or ideally prevented from forming. Air–liquid interfaces, which arise during various stages of bioprocessing, are one of the stress factors causing mAb aggregation. In this study, the behavior of an immunoglobulin G (IgG) at the air–liquid interface was investigated under flow using macro attenuated total reflection Fourier transform infrared (ATR-FTIR) spectroscopic imaging. This chemically specific imaging technique allows observation of adsorption of IgG to the air–liquid interface and detection of associated secondary structural changes. Chemical images revealed that IgG rapidly accumulated around an injected air bubble under flow at 45 °C; however, no such increase was observed at 25 °C. Analysis of the second derivative spectra of IgG at the air–liquid interface revealed changes in the protein secondary structure associated with increased intermolecular β-sheet content, indicative of aggregated IgG. The addition of 0.01% w/v polysorbate 80 (PS80) reduced the amount of IgG at the air–liquid interface in a static setup at 30 °C; however, this protective effect was lost at 45 °C. These results suggest that the presence of air–liquid interfaces under flow may be detrimental to mAb stability at elevated temperatures and demonstrate the power of ATR-FTIR spectroscopic imaging for studying the structural integrity of mAbs under bioprocessing conditions.

## Introduction

Monoclonal antibodies (mAbs) represent the largest class of biopharmaceuticals and are used for the treatment of a wide range of diseases including autoimmune and respiratory diseases, infections, and various types of cancer.^1,2^ They have a number of advantages over small-molecule drugs, including their capacity to bind to their targets with high affinity and high specificity.^3^ However, a major disadvantage of mAbs is that they require extensive bioprocessing. A typical bioprocess for the production of mAbs starts with mammalian cell culture, where the expressed mAbs are secreted into the growth medium containing various components, including cellular components, salts, nutrients, and host-cell proteins. At the harvest step, the cell culture supernatant is separated from waste material and purified through several unit operations, termed the downstream process. Generally, this includes affinity chromatography, i.e., Protein A chromatography, followed by polishing steps such as cation and/or anion exchange chromatography. Lastly, the mAb is formulated through ultrafiltration/diafiltration (UF/DF) and bulk frozen before fill and finish.^4,5^

Throughout the bioprocess, mAbs are exposed to a range of different conditions, which may pose challenges to their physical and chemical stability. For example, changes in the pH and salt concentration during the chromatography, viral inactivation, and buffer exchange steps may destabilize the protein structure and/or cause chemical modifications to the protein.^6,7^ Furthermore, interactions with a range of matrices and surfaces, such as filters, columns, and membranes, as well as physical stress and shear forces caused by sparging, pumping, mixing, and shaking, may lead to loss of the native protein structure and potentially protein function.^7,8^ During bioprocessing, aggregate levels may reach up to 30% at the cell culture stage and may be as high as 25% when eluting from the Protein A column due to the low pH conditions.^9^ In addition to the upstream and downstream processing steps, packaging, transportation, storage, and administration steps following production can also negatively impact protein stability, for example due to temperature variations, where the mAb is exposed to temperatures outside the acceptable range, unintentional freeze–thaw cycles, interfacial stresses, and exposure to light.^5,7,10−13^

The stress conditions described above may lead to partial protein unfolding and mAb aggregation. Depending on the stress conditions to which the mAb is exposed, the extent of aggregation and the type of aggregates formed may differ considerably. Moreover, the effect these aggregates have on the biological activity and potency will vary based on the source of aggregation.^9^ For example, a case study on the drug Rituximab demonstrated that for samples containing 20% aggregates, the biological potency decreased by roughly 20% for mechanically stressed samples (stirring), roughly 30% for chemically stressed samples (pH changes and oxidation), and more than 75% for samples that underwent multiple freeze–thaw cycles, based on cell-based assays.^14^

The process of protein aggregation can be described by several mechanisms or pathways, ultimately leading to the formation of soluble and/or insoluble aggregates, which may or may not precipitate. The mechanisms at play differ from protein to protein and the conditions to which the protein is exposed. In addition, multiple pathways may occur simultaneously in any given system.^15,16^ Typically, protein aggregation involves some degree of conformational change at the monomer or oligomer level, leading to irreversible formation of non-native structures.^17^

Since protein function highly depends on the protein structure, it is crucial that the mAb maintains the native fold throughout its life cycle. The bioprocessing pipeline for a given biotherapeutic should therefore be optimized to prevent/limit aggregation. Any aggregates that may have formed need to be detected and removed from the final product as they can reduce efficacy and may cause undesirable immunogenic responses in the patient.^18,19^ For example, the immune system may recognize the mAb as foreign and induce an adaptive immune response, causing the therapeutic effect to be neutralized.^20^

Although the mechanisms are not completely understood, there is substantial evidence that air–liquid interfaces induce mAb aggregation and subsequent particle formation when mAbs are exposed to them, particularly in combination with agitation.^21−25^ Air–liquid interfaces arise not only in the form of air bubbles (e.g., during sparging, pumping, mixing, and shaking) but also in the form of headspace (e.g., in storage vials, IV bags, and syringes).^11^ In this study, attenuated total reflection Fourier transform infrared (ATR-FTIR) spectroscopic imaging was used to investigate the behavior of a mAb at the air–liquid interface of air bubbles while flowing through a channel, resembling the conditions that mAbs may encounter during their production, transportation, and storage. A number of studies have been conducted on this topic using FTIR spectroscopy;^22,26−28^ however, none have used FTIR spectroscopic imaging to observe mAb aggregation and structural changes at the air–liquid interface of air bubbles under flow.

ATR-FTIR spectroscopy is a commonly used analytical tool based on the absorption of infrared light by a sample due to molecular vibrations. This chemically specific technique is particularly powerful for protein structural studies as it is nondestructive, allows study of proteins under a range of conditions (e.g., changing temperature), and yields high-quality data for samples of different forms (e.g., solids, liquids, and hydrated films). Unlike many other analytical tools, no protein labeling or complex sample preparation is required, meaning that the protein of interest can be studied under near-physiological conditions.^29−31^ For mAbs and proteins in general, the Amide I, Amide II, and Amide III bands are the most prominent in the absorption spectrum and arise from molecular vibrations in the polypeptide backbone. The Amide I band is particularly useful for protein structural analysis, as it is sensitive to changes in the secondary structure of proteins and can be more easily interpreted than the other Amide bands.^32^ By applying ATR-FTIR spectroscopic imaging, which combines the FTIR spectrometer with a focal plane array (FPA) detector, we can collect a grid of 4096 spectra simultaneously in a matter of minutes. This way, chemical information is obtained on an area of the sample, with each pixel representing an IR spectrum. By plotting the absorbance value of a specific component for each pixel, chemical images are generated allowing for the study of distributions of components within the sample and dynamic systems over time.^33^ Previously, this spectroscopic imaging approach has been successfully applied by us to the analysis of protein crystallization,^34^ protein aggregation due to thermal stress^35,36^ and freeze–thaw cycles.^37^ When combined with microfluidics, protein samples may be studied under flow, resembling bioprocessing conditions and demonstrating the applicability of this technique to in-line or online measurements.^5^

Here, we used ATR-FTIR spectroscopic imaging to obtain insights into the behavior of IgG at the air–liquid interface of air bubbles while flowing through a channel under different conditions. We observed that exposure to the air–liquid interface of air bubbles induced IgG aggregation at the interface within minutes; however, only when the protein is exposed to elevated temperatures. Interestingly, polysorbate 80, a commonly used surfactant, was able to prevent IgG from accumulating at the interface at 30 °C but lost its protective effect at 45 °C. These results indicate that air–liquid interfaces in the form of air bubbles may have a detrimental effect on mAb stability, particularly in combination with elevated temperatures.

## Experimental Section

### Sample Preparation

Immunoglobulin G mAbs were produced in Chinese hamster ovary (CHO) cell cultures and purified through several downstream processing steps. Cell culture supernatant samples containing the mAb were provided by Cleo Kontoravdi and stored at −20 °C prior to purification. After defrosting at 4 °C, the samples were centrifuged at 3000*g* for 10 min and filtered through a 0.45 μm disk filter to remove any remaining cell debris, large particles, and/or aggregates. Using a HiPrep desalting column (GE Healthcare), low-molecular-weight contaminants and salts were removed, and the sample was exchanged into 50 mM phosphate/150 mM NaCl buffer, pH 7.4. Following this, affinity chromatography was performed using a 4.7 mL MabSelect protein A column (Cytiva) equilibrated with phosphate buffer at pH 7.4. The mAb was eluted from the column with a 0.1 M sodium citrate buffer at pH 3.0–3.6 into a collection tube containing 1 M TRIS-HCl, pH 9.0. The ratio of TRIS-HCl buffer to the eluted sample was 1:5 v/v for each collected fraction. The eluted samples were analyzed by sodium dodecyl sulfate-polyacrylamide gel electrophoresis to confirm the purity of the mAb prior to exchange into phosphate buffer at pH 7.4 using 50 kDa molecular weight cutoff filters. The mAb sample was concentrated to 10 mg/mL, divided into aliquots and snap-frozen using liquid N_2_, before storage at −80 °C. For the static experiments with samples containing 0.01% w/v polysorbate 80 (PS80), polysorbate 80 at 10% w/v was diluted in phosphate buffer prior to addition to the IgG sample. For the control IgG sample containing 0.0% PS80, the same volume of phosphate buffer was added, ensuring the same concentration of protein in the test and control samples.

### ATR-FTIR Spectroscopic Imaging

Macro ATR-FTIR spectroscopic imaging was performed using a Tensor 27 spectrometer (Bruker, U.K.) coupled to an IMAC large sample compartment (Bruker, U.K.) and a single reflection variable angle ATR accessory (Pike Technologies, Madison, WI) or a fixed angle of incidence (45°) accessory with a heated ZnSe internal reflection element (IRE) (Specac). The IRE was heated to the desired temperature with a nichrome-wire-based heating controller. An MCT FPA detector with 64 × 64 pixels was employed, with a pixel size of 40 × 40 μm^2^. With this setup, 4096 spectra were recorded simultaneously in the continuous scan mode over the range of 900–3900 cm^–1^. Spectra were recorded by coadding 32 scans at a resolution of 4 cm^–1^. For the flow setup, a PDMS channel of dimensions 7 mm × 2 mm × 1 mm was held in position and secured onto the ZnSe IRE by using a PMMA top plate. The channel was connected to a syringe with needle (inlet) and waste container (outlet) with PTFE tubing with a 0.5 mm inner diameter. The syringe was then placed in a syringe pump (Harvard Apparatus) with the flow controlled at 10 μL/min. An overview of the experimental setup is presented in Figure S1. For the static experiments, the same setup was used, but the syringe containing the buffer or IgG sample was not connected to the pump.

### Experimental Procedure

For the IgG flow experiments, phosphate buffer (pH 7.4) and protein solution of 10 mg/mL were flowed sequentially through the PDMS channel at a flow rate of 10 μL/min, while measurements were taken at a 45° angle. Measurements were taken at room temperature (RT) after which the ZnSe IRE was heated to 25 or 45 °C and measurements were taken at intervals of 5 min over 40 min of heating. For the air–liquid interfacial stress experiments, an air bubble was introduced into the channel using a syringe and a needle after the RT measurements finished. All experiments were conducted in triplicate. For the static experiments with and without 0.01% w/v PS80, the fixed angle of incidence accessory was used (45° angle), and measurements were taken every 3 min over 30 min of heating at 30 °C.

### Data Analysis

Chemical images were generated by plotting the integrated absorbance of the Amide I band (1700–1600 cm^–1^) or the Amide II band (1580–1490 cm^–1^) for each pixel. Average spectra were extracted from the areas of interest, followed by buffer and water vapor subtraction using OPUS (Bruker Technologies). Second derivative spectra were generated in OPUS (Bruker Technologies) and further spectral processing, including baseline subtraction and normalization of the Amide I band was performed in OriginPro (OriginLab, Northampton, MA) and/or MATLAB (MathWorks, Natick, MA).

## Results and Discussion

### ATR-FTIR Spectroscopic Imaging of IgG Adsorption to Air–Liquid Interfaces under Flow

**Figure 1:**
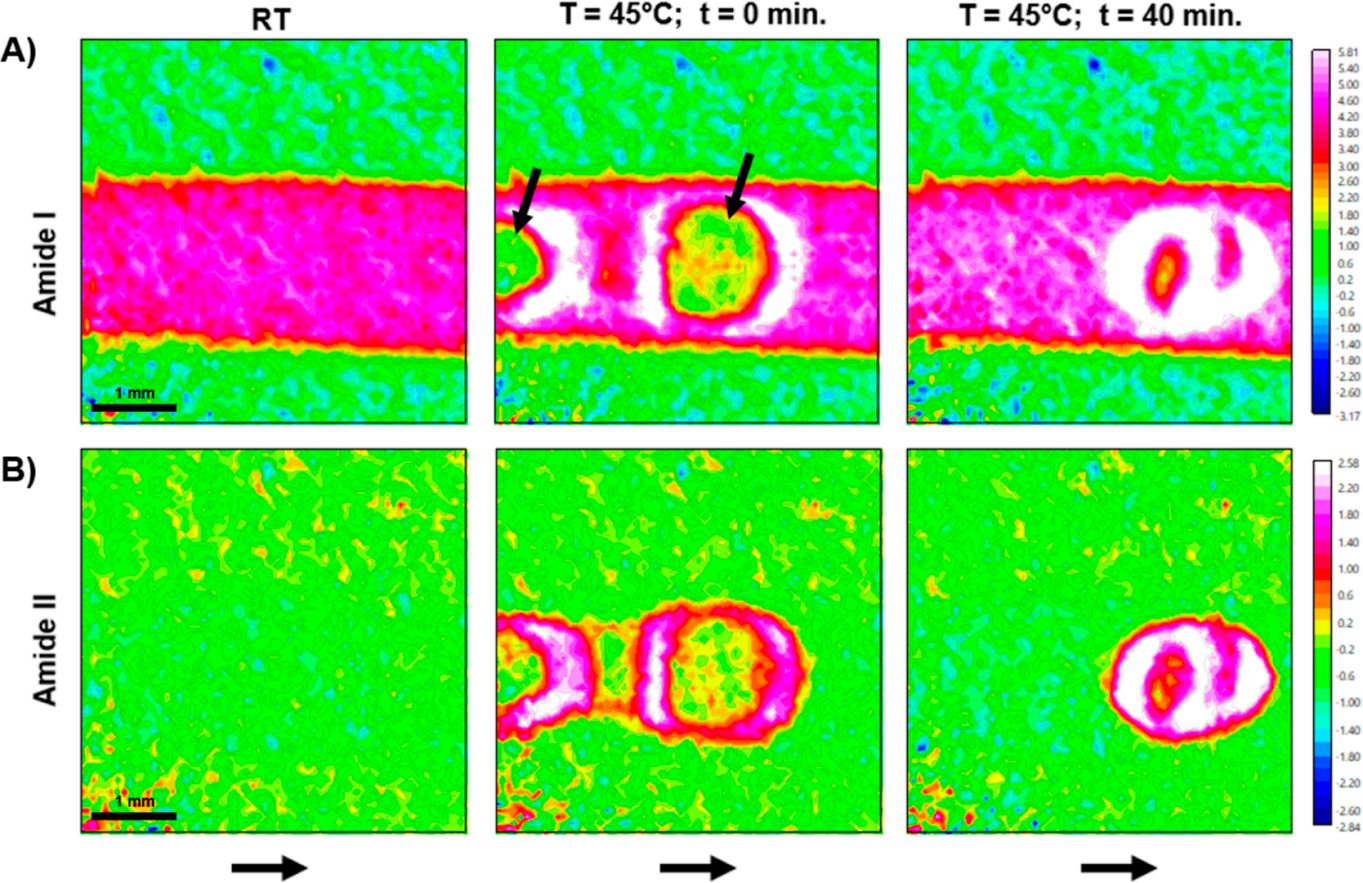
Chemical images of a 45 °C flow experiment with the presence of air bubbles. (A) Chemical images were obtained by integrating over the Amide I band (1700–1600 cm^–1^) of the spectra measured before air bubble injection at RT, right after air bubble injection and heating to 45 °C (*T* = 45 °C; *t* = 0 min), and after 40 min of heating (*T* = 45 °C; *t* = 40 min). (B) Chemical images obtained by integrating over the Amide II band (1580–1490 cm^–1^). The arrows in the top image indicate the areas of low absorbance corresponding to the air bubble; the arrows pointing to the right indicate the direction of flow.

ATR-FTIR spectroscopic imaging was applied to study the behavior of IgG at the air–liquid interface of air bubbles under flow at elevated temperature. Experiments were conducted under flow to more accurately capture the stress encountered by the mAbs during bioprocessing, where the protein is pumped through tubing and mixed in vessels at various points in the process. The effect of exposure to an air–liquid interface was compared for two different temperatures, 25 and 45 °C. These temperatures were selected to represent accelerated and stressed conditions, which mAbs may be exposed to throughout their life cycle, particularly during transport and storage.^11^ Furthermore, studying mAbs under accelerated conditions in our experimental setup may provide information on the long-term stability of mAbs under less stressing conditions.^35^ Flow experiments at both temperatures without the introduction of an air bubble were used as controls. In bioprocessing, the mAb concentration can range from 1 g/L to more than 20 g/L depending on the processing step. The final formulation of therapeutic mAbs is typically at a higher concentration (>100 mg/mL).^38,39^ A concentration of 10 mg/mL was selected for the flow experiments, as this concentration lies within the range of concentrations in which a mAb could be in during production. Chemical images were obtained by plotting the integrated absorbance of the Amide I band (1700–1600 cm^–1^) and the Amide II band (1580–1490 cm^–1^) for all pixels. In Figure 1, the chemical image of the RT measurement prior to heating and air-injection is shown, as well as the chemical images of the heated sample immediately after air-injection (*t* = 0 measurement), and 40 min of heating at 45 °C. As the Amide I band overlaps with the spectral band of the bending mode of water, both water and protein absorbance are represented by the 1700–1600 cm^–1^ integration range and therefore both contribute to the absorbance in the chemical images (Figure 1, top). However, the Amide II band does not overlap with the spectrum of water, and integration over this band results in images showing high absorbance only at areas of high protein concentration near the measuring surface of the IRE (Figure 1, bottom). Air is injected just before the *t* = 0 measurement and can be observed by the circular areas of low absorbance within the channel (black arrows).

From the chemical images of the 45 °C experiment (Figure 1), it can be clearly seen that areas of high Amide I and Amide II absorbance, i.e., white areas, first appear around the air bubble at time point 0 and increase over time. The strong increase in absorbance upon air-injection suggests that the mAbs are assembling at and/or adsorbing to the air–liquid interface, resulting in detection of high protein concentration around the air bubble. Interestingly, for the experiment at 45 °C without air–liquid interface, no areas of high Amide I and Amide II absorbance were observed within the channel (images not shown), confirming that the air–liquid interface is key to the local accumulation of IgG. However, a slight increase in overall Amide I absorbance could be observed compared to the RT measurement taken prior to heating to 45 °C. Thermal denaturation is expected to be limited since the melting temperature of IgG is much higher; however, heating the sample to 45 °C may stress the protein sufficiently to cause some degree of unfolding.^35^ On the contrary, at 25 °C under flow, the Amide I and Amide II absorbance did not increase upon air injection nor after 40 min of flow at 25 °C (Figure S2). Similarly, the 25 °C flow experiment without air injection did not result in an increase in Amide I or Amide II absorbance. These findings were consistent between replicates and suggest that it is a combination of temperature and the presence of air–liquid interfaces that induces the formation of areas of increased protein concentration. Although the exact size of the injected air bubble could not be controlled, the trend observed for each condition tested was consistent between replicates. A constant bubble size or surface area would be ideal, as it would reduce variability between experiments. However, based on all the data that was obtained during this study, it can be concluded that slight differences in bubble size do not necessarily affect the extent of aggregation (smaller bubbles in the channel would still result in areas of high Amide I and Amide II absorbance close to the interface). Future work may focus on controlling the interfacial area to study the effect of increasing/decreasing surface area on the extent of protein aggregation.

In order to quantify the increase in Amide I absorbance and allow for analysis of protein secondary structure, average spectra were extracted from the areas of high absorbance adjacent to the air bubble for each time point (minimum of 112 pixels averaged for all replicates to obtain a good S/N). When no increase in absorbance was observed in the chemical images, i.e., for the 25 °C flow experiment, an average spectrum was extracted from an area of similar size near the air–liquid interface. The average protein spectra were then corrected for water vapor using a water vapor spectrum and buffer-subtracted using the average spectrum from the phosphate buffer measurement taken at the start of each experiment.

**Figure 2:**
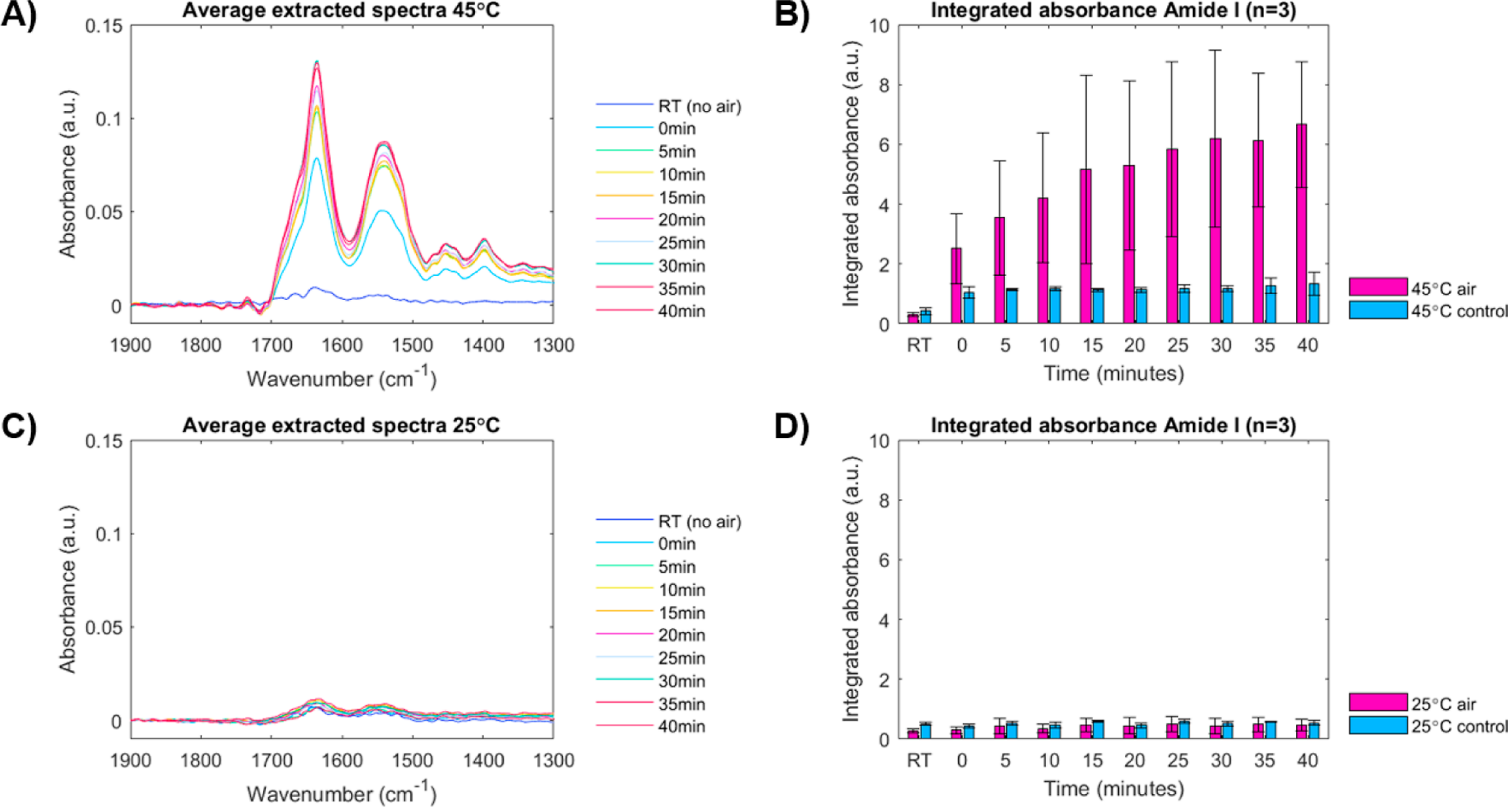
Quantification of the Amide I band from chemical images. (A,C) Nine-point smoothed (Savitzky-Golay) spectra extracted from areas near the air–liquid interface during 25 and 45 °C flow experiments plotted for all time points (data of one experiment presented). (B,D) Integrated Amide I absorbance plotted over time for 25 and 45 °C flow experiments with and without air (*n* = 3 for each condition).

The water vapor and buffer-subtracted protein spectra corresponding to the above shown chemical images are presented in Figure 2A,C for the 45 and 25 °C flow experiments, respectively. By calculating the area under the Amide I band, the increase in absorbance near the air bubble could be quantified and plotted as a function of time, as shown in Figure 2B,D. At 25 °C, no increase in Amide I absorbance was observed over time, and the integrated absorbance remained the same, regardless of the presence of an air–liquid interface (Figure 2C,D). However, at 45 °C, there is a clear increase in Amide I absorbance over time when an air bubble is present in the channel (*p* < 0.05 at *t* = 40 min), as was observed from the chemical images shown in Figure 1. A comparable trend was observed for the Amide II absorbance, which was quantified by integrating over 1580–1490 cm^–1^ (Figure S3). In the absence of an air bubble in the channel, the Amide I absorbance increases at the start of heating to 45 °C and remains stable over time (blue bars in Figure 2B). This increase is small, but significant when compared to the 25 °C flow experiment (*p* < 0.05).

### Secondary Structural Changes in IgG at the Air–Liquid Interface

From the chemical images and extracted spectra, it was found that the combination of elevated temperature (45 °C) and the presence of an air bubble results in significant increases in protein concentration near the air–liquid interface. The data presented show that within a time frame of minutes, IgG starts to adsorb to and accumulate at the interface, while the area of high Amide I absorbance expands outward over time. By analyzing the changes in the Amide I band of IgG at the air–liquid interface compared to native IgG in solution, and comparing this to highly aggregated IgG at 80 °C, we aim to understand whether IgG is undergoing secondary structural changes at the air–liquid interface. Here, the second derivative spectrum of IgG exposed to extreme heat (80 °C) is used as a reference for highly aggregated IgG, as it is known that the protein loses its native structure and aggregates at this temperature.^26,40^

Since the structural components underlying the Amide I band overlap and cannot be identified directly from the average extracted spectra, the second derivative spectra were analyzed.

First, the normalized second derivative spectra of the Amide I band of IgG at the air–liquid interface after *t* = 0 and *t* = 40 min of heating were compared (Figure S4). Minimal changes to the main peak at 1634 cm^–1^ (β-sheets) were observed between the *t* = 0 and *t* = 40 min measurements, suggesting that the protein structure at the interface remains largely the same while heating at 45 °C. The time of heating therefore does not seem to have a strong effect on the main structure of IgG at the air–liquid interface. However, small differences at other wavenumber ranges were observed between the two time points, including a decrease at 1644 cm^–1^ (unordered structures) and a small increase at 1662 cm^–1^ (β-turn).^41,42^

**Figure 3:**
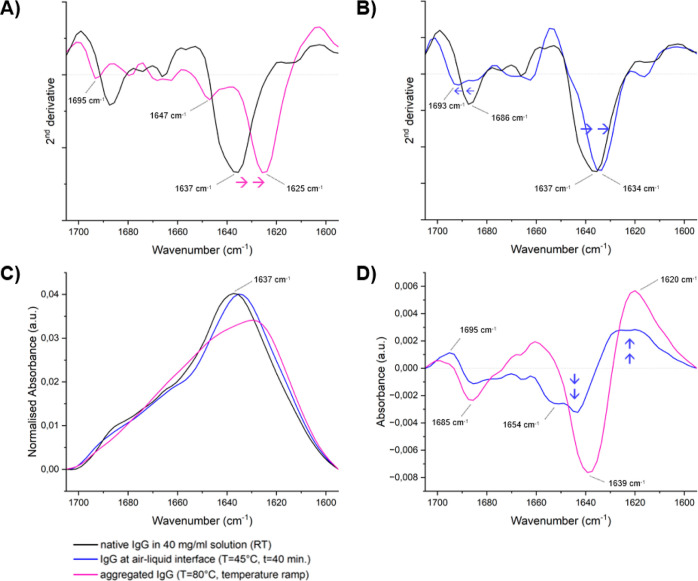
Structural analysis of the Amide I band of IgG. (A) Normalized second derivative spectra of heat-stressed IgG (80 °C) and 40 mg/mL native IgG in solution at RT (average of three replicates). (B) Normalized second derivative spectra of IgG at the air–liquid interface (45 °C, *t* = 40 min) and native IgG in solution at RT (average of three replicates). (C) Normalized Amide I absorbance (average of three replicates) of native IgG in solution at RT, IgG at the air–liquid interface (45 °C, *t* = 40 min) and heat-stressed IgG (80 °C). (D) Difference spectra of the Amide I band for IgG at the air–liquid interface (45 °C, *t* = 40 min) and heat-stressed IgG (80 °C). Blue arrows indicate main structural changes.

Furthermore, the normalized second derivative spectra of heat-stressed IgG (80 °C) and IgG at the air–liquid interface (45 °C, *t* = 40 min) were compared to native IgG in solution at RT, respectively. The heat-stressed IgG spectrum, measured as a part of a temperature ramp experiment and used as a reference for non-native aggregated IgG, shows a clear shift in peak position from 1637 to 1625 cm^–1^ (Figure 3A), in agreement with previously published results.^26,43^ This shift can be assigned to a loss of intramolecular β-sheet structure and an associated increase in intermolecular β-sheet structure as a result of aggregate formation. Furthermore, a peak at 1647 cm^–1^ is observed, which can be assigned to disordered structures.^42^ Subsequently, the normalized second derivative spectrum of IgG at the air–liquid interface was plotted together with the native IgG second derivative spectrum in Figure 3B (for both graphs the average of three replicates was plotted). Here, it can be observed that, although small, there is a shift in the main peak position from 1637 to 1634 cm^–1^, indicating a loss of intramolecular β-sheet structure. Based on the previous comparison between the *t* = 0 and *t* = 40 min measurements (Figure S4), it can be concluded that this shift of the main Amide I peak appears right at the start of heating at 45 °C rather than developing over time. Lastly, the peak at 1686 cm^–1^ in the native IgG spectrum has largely disappeared and is replaced by a small peak around 1693 cm^–1^ (Figure 3B), indicating an increase in intermolecular β-sheet structures.^43,44^

The changes in the secondary structure were also evaluated by plotting difference spectra for the two stress conditions. Difference spectra were obtained by subtracting the normalized Amide I band of native IgG in solution (RT) from the Amide I bands of interest, i.e., IgG at the air–liquid interface (45 °C, *t* = 40 min) and heat-stressed IgG (80 °C) as a reference for highly aggregated protein. The normalized Amide I bands are plotted in Figure 3C (average of three replicates) and the difference spectra obtained after subtraction are plotted in Figure 3D (average of three replicates). By comparing the overall shape of the Amide I bands it can be confirmed that native IgG in solution (black line) consists of mainly intramolecular β-sheet structure (peak at 1637 cm^–1^) characteristic of IgG and that IgG at the air–liquid interface (blue line) largely maintains this structure.^26,35^ From Figure 3C it can also be observed that heating at 80 °C results in significant structural changes (magenta line); however, by plotting the difference spectrum, it is possible to determine which specific wavenumber ranges are affected the most in this case. The difference spectra in Figure 3D show that for the highly aggregated IgG at 80 °C, the intramolecular β-sheet structure is replaced by a mostly intermolecular β-sheet structure, represented by the negative peak around 1637 cm^–1^ and the positive peak around 1620 cm^–1^, respectively. Similarly, the difference spectrum of IgG at the air–liquid interface reveals that intermolecular β-sheet structures are gained and intramolecular β-sheet structures are lost. Moreover, the difference spectrum shows that IgG at the air–liquid interface loses β-turns (1685–1666 cm^–1^), unordered structures (1647 cm^–1^), and α-helical structures (1654 cm^–1^). Lastly, a gain in β-sheet structures is observed around 1695 cm^–1^ compared to that of native IgG at RT.

From these results, it can be concluded that IgG undergoes small structural changes at the air–liquid interface at 45 °C, indicating that aggregation is promoted by exposure to air–liquid interfaces at elevated temperatures. Although the temperatures during mAb production are generally strictly controlled, studying the mAb stability at elevated temperatures is very relevant. For example, accelerated stability studies evaluate long-term protein stability based on exposure to elevated temperatures for shorter periods of time.^45^ Furthermore, during transportation, storage at local hospitals and pharmacies, and handling by the patient/caretaker, biopharmaceuticals may be exposed to undesirably high temperatures outside of the intended range.^11^

### Effect of Polysorbate 80 Addition on IgG Adsorption to Air–Liquid Interface in Static System

As demonstrated in the previous section, exposure to air–liquid interfaces of air bubbles under flow stimulates mAb aggregation at a temperature of 45 °C. Due to the loss of therapeutic function and undesirable immunogenic responses related to mAb aggregates, this phenomenon should be minimized. In biopharmaceutical development excipients, particularly polysorbate 20 and polysorbate 80, are commonly added to the formulation to prevent interfacial interactions and limit protein–protein interactions.^24,46^ Typically, these surfactants are added to the formulation after the UF/DF step and before the bulk freezing step, over a concentration range of 0.003%–0.8% w/v.^47,48^ Polysorbates are nonionic surfactants which may act via two proposed mechanisms: (i) competition for adsorption to the interface between the mAb and the polysorbate and (ii) the binding of polysorbate to the mAbs with low affinity, resulting in different interactions between mAbs.^47^ In this study, polysorbate 80 (PS80 or Tween 80) was added to the mAb solution, and its capability of preventing protein adsorption and subsequent aggregation at the air–liquid interface was assessed using ATR-FTIR spectroscopic imaging.

**Figure 4:**
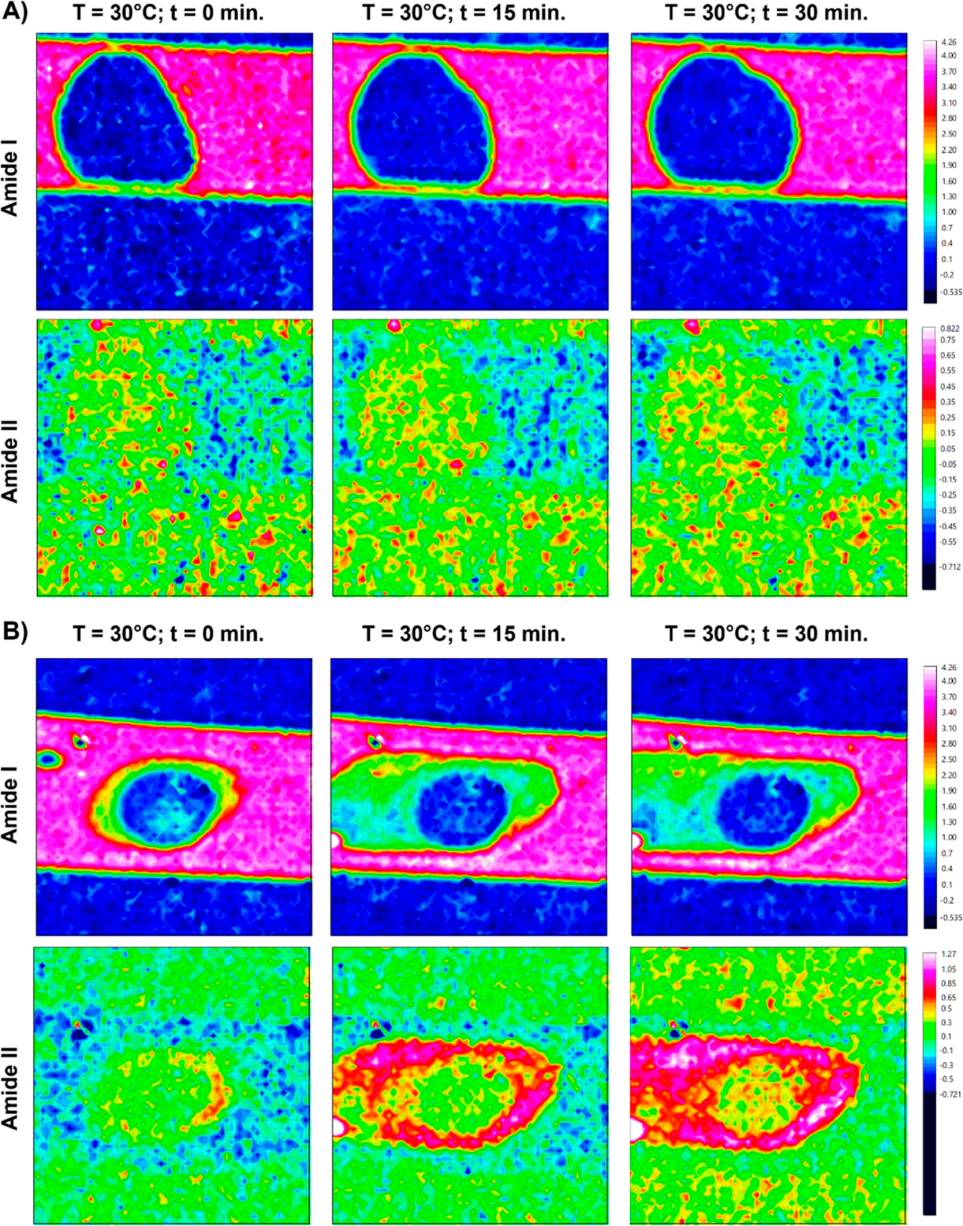
Chemical images of 30 °C static experiments with the presence of air bubbles with and without PS80 at 0.01% w/v. (A) IgG sample with 0.01% w/v PS80. Measurements at *t* = 0 min, *t* = 15 min, and *t* = 30 min of heating at 30 °C. (B) IgG sample without PS80. Measurements at *t* = 0 min, *t* = 15 min, and *t* = 30 min of heating at 30 °C.

PS80 concentrations of 0.001%, 0.01%, 0.1%, and 1% w/v were tested with a mAb concentration of 10 mg/mL. Initially, flow experiments at 45 °C were conducted with air-injection in the same way as before; however, none of the PS80 additions resulted in preventing IgG from accumulating at the interface (Figure S5). In order to assess the effectiveness of adding PS80 better, the experiment was adjusted to a static setup (i.e., no flow in the channel during measurements) at a lower temperature (30 °C) and at a single PS80 concentration (0.01% w/v) (Figure 4). Under these conditions, mAbs still accumulated at the air–liquid interface in the absence of PS80, albeit to a lesser extent compared with 45 °C heating (Figure 4B). In addition, in the static setup, the air bubble is less likely to move within the channel, making spectral extraction from the chemical images easier and reproducibility higher. In Figure 4, the chemical images of one set of static experiments with and without 0.01% w/v PS80 addition are presented for three time points (*t* = 0 min, *t* = 15 min, and *t* = 30 min). Figure 4A clearly shows that no protein is adsorbing to or accumulating at the air–liquid interface of the injected air bubble when PS80 is present, whereas Figure 4B clearly shows the opposite for the sample without a surfactant.

**Figure 5:**
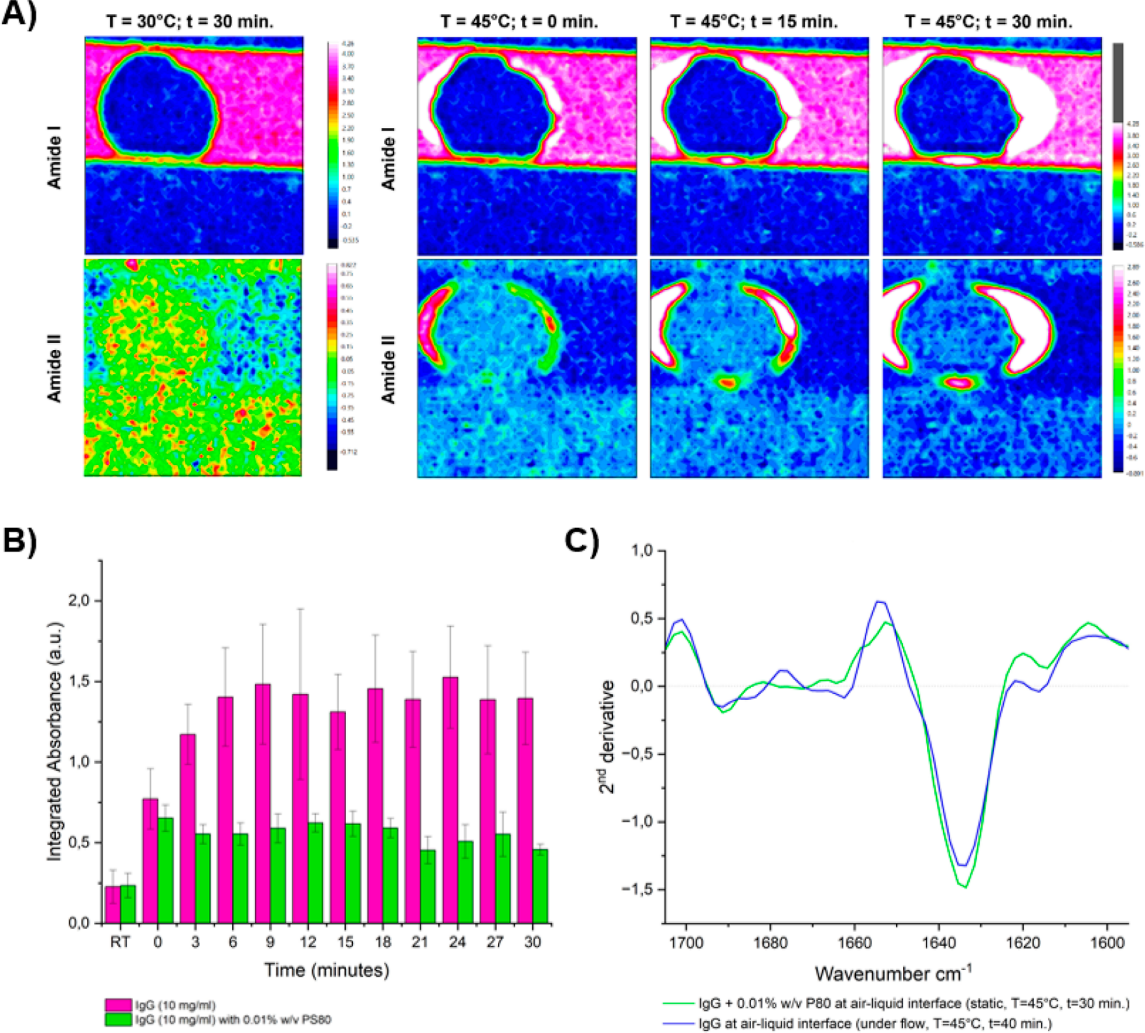
Addition of 0.01% PS80 to reduce IgG aggregation at the air–liquid interface. (A) Chemical images of *t* = 30 min static experiment at 30 °C and subsequent increase to 45 °C with the presence of an air bubble for 10 mg/mL IgG + 0.01% w/v PS80. (B) Quantification of Amide I absorbance near the air–liquid interface from chemical images obtained from the 30 °C static experiments. Integrated Amide I absorbance plotted over time for 0.0% PS80 and 0.01% w/v PS80 (*n* = 3). (C) Normalized average second derivative spectra of IgG in the presence of 0.01% w/v PS80 (static, *T* = 45 °C, *t* = 30 min) and in the absence of PS80 (under flow, *T* = 45 °C, *t* = 40 min).

The Amide I absorbance near the air–liquid interface was quantified for both samples by extracting average spectra from areas near the interface, subtracting the buffer and water vapor spectrum, and integrating over the Amide I band. The results of three repeats are presented in Figure 5B, which shows a 2-fold increase in Amide I absorbance near the air–liquid interface when PS80 is not added. These findings indicate that PS80 at a concentration of 0.01% w/v does have a protective effect on IgG adsorbing to the air–liquid interface at 30 °C. The exact mechanism by which polysorbates protect proteins from adsorbing to and aggregating at the air–liquid interface is still under debate; however, several studies have shown this effect.^21,25,49,50^ Interestingly, as soon as the temperature was increased from 30 to 45 °C, this protective effect was lost (Figure 5A). Within a few minutes, an area of higher protein concentration formed around the air bubble, and over time, this area expanded outward. The temperature-dependency observed here may be related to both the mAb’s instability at elevated temperatures, as well as the potential degradation of PS80.^51,52^ When comparing the second derivative spectrum of the aggregated IgG in the presence of 0.01% w/v PS80 (static, *T* = 45 °C, *t* = 30 min) to the second derivative spectrum of aggregated IgG in the absence of PS80 (under flow, *T* = 45 °C, *t* = 40 min), we observe that the spectra are very similar (Figure 5C). From this comparison, it can be concluded that the structural changes that IgG undergoes under these conditions are not changed by the addition of 0.01% w/v PS80.

Several studies have pointed out the correlation between the surface activity and interfacial stability of mAbs. Nonionic surfactants like PS80 generally have a higher surface activity compared to mAbs and therefore tend to adsorb more readily to hydrophobic surfaces, like the air–liquid interface.^23,53^ The ability of PS80 to prevent mAb adsorption to air–liquid interfaces and subsequent particle formation has been shown to depend on various factors, including the interfacial properties of the mAb under study, the surfactant concentration, and the grade of PS80 used.^50,54^ Furthermore, as demonstrated in this study, the temperature may play an important role in the interfacial stability of mAbs. The interfacial properties of mAbs, including surface activity, are likely to change with temperature.^55^ Here, it was found that the protective effect of PS80 was lost after changing the temperature from 30 to 45 °C, with mAbs rapidly adsorbing to the air–liquid interface after the temperature increase.

## Conclusions

In this study, ATR-FTIR spectroscopic imaging was applied for the first time to study protein aggregation at the air–liquid interface of air bubbles. Chemical images allowed analysis of specific areas within the flow channel and revealed that protein accumulated in high concentration around the injected air bubbles while heating at 45 °C. Only by implementing this ATR-FTIR spectroscopic imaging approach could both the distribution of protein be visualized and the associated protein structural changes revealed. It was found that temperature is a key factor in causing IgG aggregation at the air–liquid interface, with 25 °C resulting in no significant increase in Amide I absorbance at the air–liquid interface compared to a strong increase in Amide I absorbance when heating at 45 °C for 40 min. In addition to the increase in IgG concentration at the air bubble interface, the Amide I band shifted to a lower wavenumber, indicative of a gain in intermolecular β-sheet structures and aggregate formation. Furthermore, the addition of 0.01% w/v PS80, a well-known surfactant, was found to reduce the accumulation of IgG at the air–liquid interface 2-fold upon heating at 30 °C in a static system compared to a control with no PS80. However, increasing the temperature of the solution to 45 °C immediately resulted in protein aggregation, demonstrating the limitations of this surfactant at an elevated temperature. The methods presented here can be applied to other biopharmaceuticals and could be used as part of the quality control checks at formulation development or as an in-line or online monitoring technique during downstream bioprocessing. Specifically, the use of small-scale flow devices in ATR-FTIR spectroscopic imaging measurements, as demonstrated in this study, shows how biopharmaceuticals could be analyzed and monitored under flow and under realistic bioprocessing conditions.

## Supporting Information Available

Overview of the experimental setup (Figure S1), chemical images of 25 °C flow experiment with the presence of air bubbles (Figure S2), comparison of Amide I and Amide II integrated absorbance for 45 °C flow experiments with the air–liquid interface (Figure S3), second derivative spectra of the Amide I band of IgG at the air–liquid interface, comparison between *t* = 0 and *t* = 40 min of heating at 45 °C (Figure S4), and chemical images of additional flow experiments of IgG + different PS80 concentrations (Figure S5) (PDF)
