# Supplementary material for: Study of Monoclonal Antibody Aggregation at the Air–Liquid Interface under Flow by ATR-FTIR Spectroscopic Imaging
Source: Langmuir. 2024 Mar 6;40(11):5858–68. doi: 10.1021/acs.langmuir.3c03730 (PMC10956494; doi:10.1021/acs.langmuir.3c03730)
Supplement: Supplementary file 1 — la3c03730_si_001.pdf [file la3c03730_si_001.pdf]

Supplementary Information

Study of monoclonal antibody aggregation at the air-liquid interface under flow by ATR-FTIR spectroscopic imaging

Céline van Haaren[1], Bernadette Byrne[2]*, Sergei G Kazarian[1]*
[1] Department of Chemical Engineering, Imperial College London, UK
Email: s.kazarian@imperial.ac.uk
[2] Department of Life Sciences, Imperial College London, UK
Email: b.byrne@imperial.ac.uk

Table of contents

1. Overview of experimental setup **(Figure S1)**
2. Chemical images of 25°C flow experiment with the presence of air bubbles **(Figure S2)**
3. Comparison of Amide I and Amide II integrated absorbance for 45°C flow experiments with air-liquid interface. **(Figure S3)**
4. Second derivative spectra of Amide I band of IgG at air-liquid interface, comparison between t=0 and t=40 min. of heating at 45°C **(Figure S4)**
5. Chemical images of additional flow experiments of IgG + different PS80 concentrations **(Figure S5)**

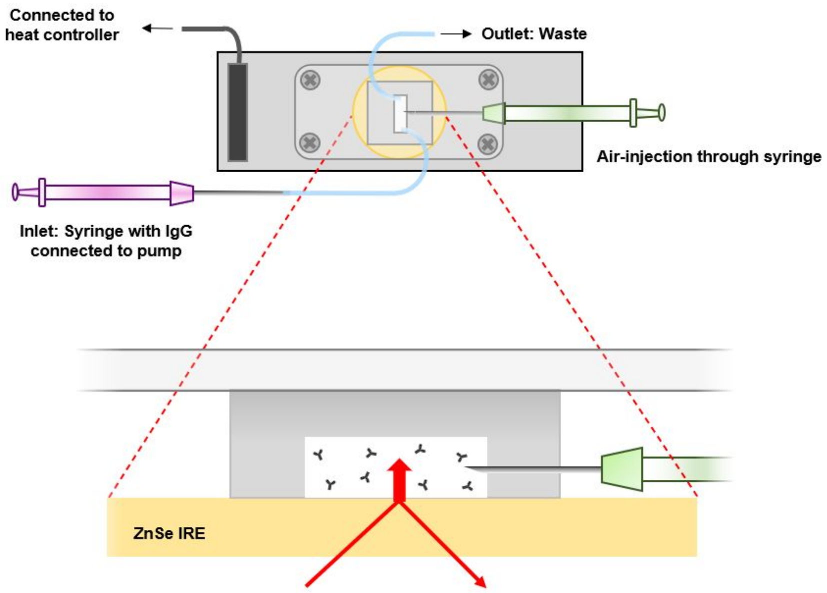

Figure S1. Overview of experimental setup. Top: top view of ATR accessory with flow channel, including syringes for IgG flow and air injection. Bottom: side view of PDMS flow channel secured onto ZnSe IRE with top plate and syringe.

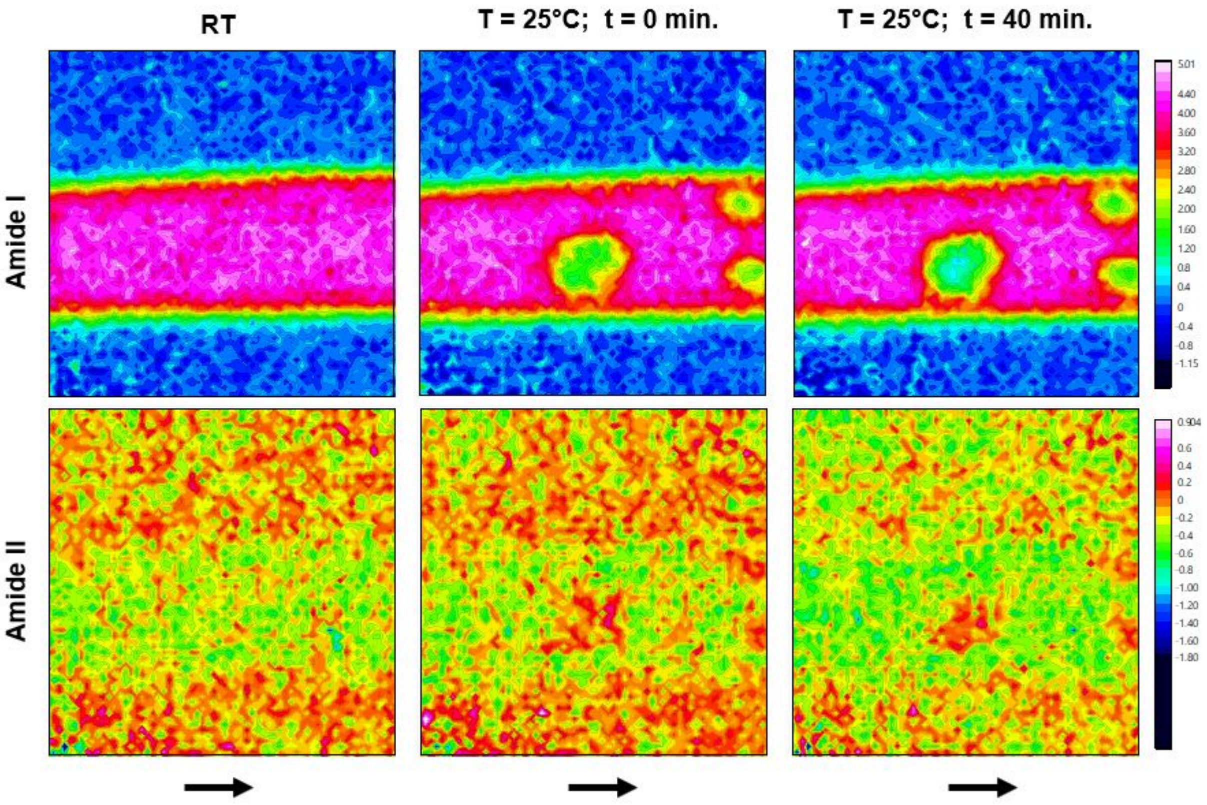

Figure S2. Chemical images of 25°C flow experiment with the presence of air bubbles.

Top: chemical images obtained by integrating over the Amide I band (1700 – 1600 cm^{-1}). Bottom: chemical images obtained by integrating over the Amide II band (1580 – 1490 cm^{-1}). The black arrows indicate the direction of flow.

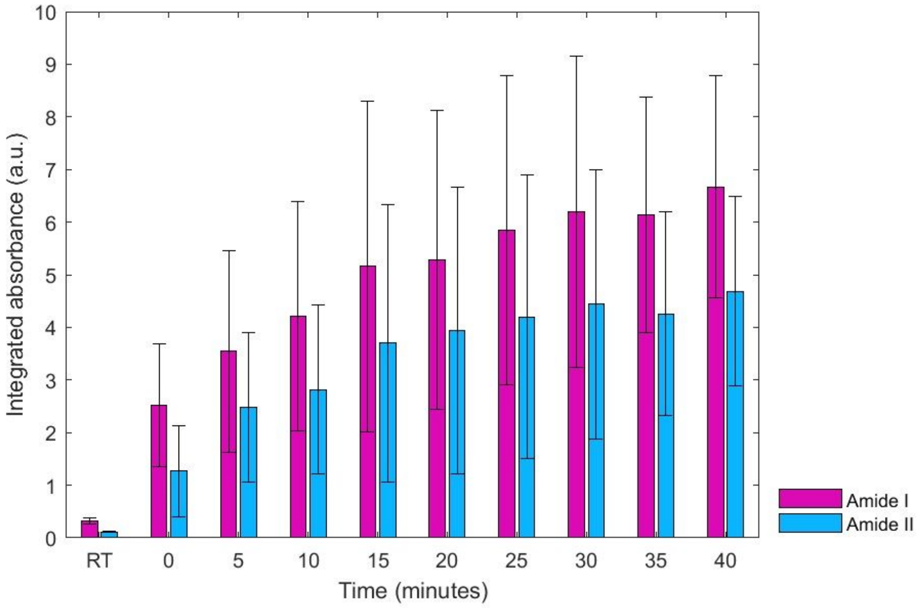

Figure S3. Comparison of Amide I and Amide II absorbance near air-liquid interface for 45°C flow experiments. Integrated absorbance of Amide I and Amide II band for flow experiments with injection of air bubble (n=3).

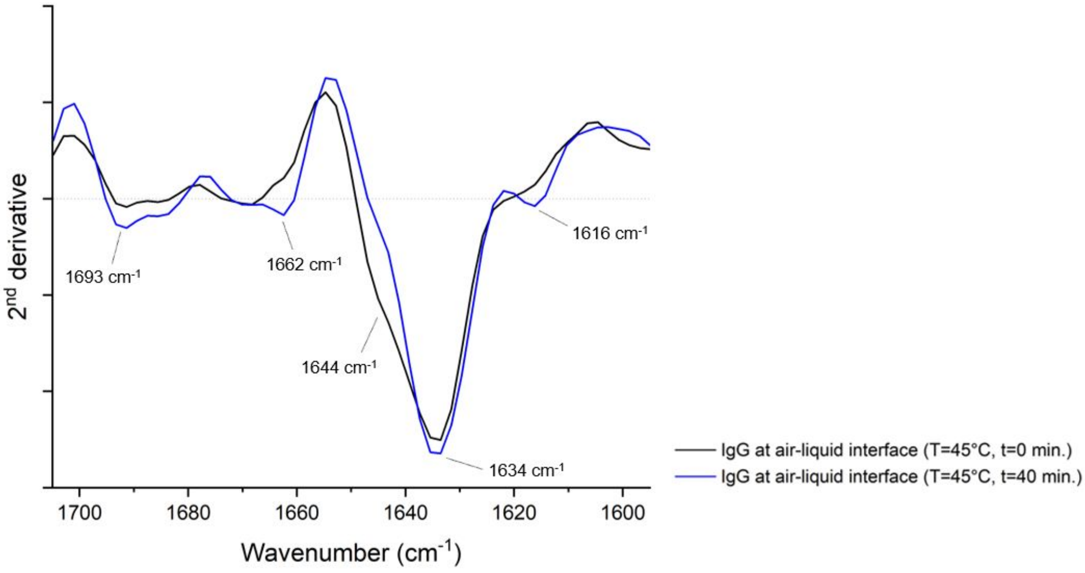

Figure S4. Normalised second derivative spectra of Amide I band.
Normalised second derivative spectra of spectra extracted from the area near air-liquid interface after t=0 and t=40 minutes of heating to 45°C (average of three replicates).

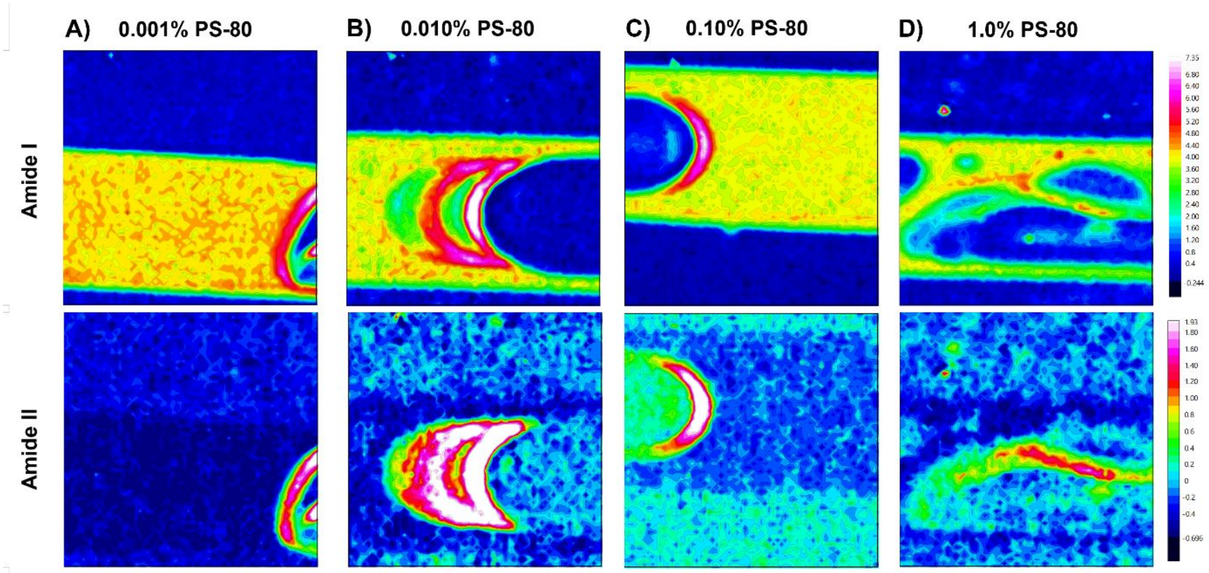

Figure S5. Chemical images of 45°C flow experiment with the presence of air bubbles at different PS80 concentrations after 15 minutes of heating. A) 0.001% w/v, B) 0.010% w/v, C) 0.10 % w/v, D) 1.0% w/v.
